# Cigarette Smoking-Induced Cardiac Hypertrophy, Vascular Inflammation and Injury Are Attenuated by Antioxidant Supplementation in an Animal Model

**DOI:** 10.3389/fphar.2016.00397

**Published:** 2016-11-09

**Authors:** Moustafa Al Hariri, Kazem Zibara, Wissam Farhat, Yasmine Hashem, Nadia Soudani, Farah Al Ibrahim, Eva Hamade, Asad Zeidan, Ahmad Husari, Firas Kobeissy

**Affiliations:** ^1^Department of Biochemistry and Molecular Genetics, Faculty of Medicine, American University of BeirutBeirut, Lebanon; ^2^ER045, PRASE, DSST, Lebanese UniversityBeirut, Lebanon; ^3^Laboratory of Cardiovascular Diseases and Stem Cells, Biochemistry Department, Faculty of Sciences-1, EDST, Lebanese UniversityBeirut, Lebanon; ^4^Department of Physiology, Faculty of Medicine, American University of BeirutBeirut, Lebanon; ^5^Department of Biology, Faculty of Sciences, EDST, Lebanese UniversityHadath, Lebanon; ^6^Division of Pulmonary and Critical Care Medicine, Department of Internal Medicine, American University of BeirutBeirut, Lebanon

**Keywords:** cigarette smoking, pomegranate juice, inflammation, hypertrophy, calcification, reactive oxygen species, cardiovascular diseases

## Abstract

**Background:** Cardiovascular diseases are the leading causes of morbidity and mortality worldwide. Cigarette smoking remains a global health epidemic with associated detrimental effects on the cardiovascular system. In this work, we investigated the effects of cigarette smoke exposure on cardiovascular system in an animal model. The study then evaluated the effects of antioxidants (AO), represented by pomegranate juice, on cigarette smoke induced cardiovascular injury. This study aims at evaluating the effect of pomegranate juice supplementation on the cardiovascular system of an experimental rat model of smoke exposure.

**Methods:** Adult rats were divided into four different groups: Control, Cigarette smoking (CS), AO, and CS + AO. Cigarette smoke exposure was for 4 weeks (5 days of exposure/week) and AO group received pomegranate juice while other groups received placebo. Assessment of cardiovascular injury was documented by assessing different parameters of cardiovascular injury mediators including: (1) cardiac hypertrophy, (2) oxidative stress, (3) expression of inflammatory markers, (4) expression of Bradykinin receptor 1 (Bdkrb1), Bradykinin receptor 2 (Bdkrb2), and (5) altered expression of fibrotic/atherogenic markers [(Fibronectin (Fn1) and leptin receptor (ObR))].

**Results:** Data from this work demonstrated that cigarette smoke exposure induced cardiac hypertrophy, which was reduced upon administration of pomegranate in CS + AO group. Cigarette smoke exposure was associated with elevation in oxidative stress, significant increase in the expression of IL-1β, TNFα, Fn1, and ObR in rat's aorta. In addition, an increase in aortic calcification was observed after 1 month of cigarette smoke exposure. Furthermore, cigarette smoke induced a significant up regulation in Bdkrb1 expression level. Finally, pomegranate supplementation exhibited cardiovascular protection assessed by the above findings and partly contributed to ameliorating cardiac hypertrophy in cigarette smoke exposed animals.

**Conclusion:** Findings from this work showed that cigarette smoking exposure is associated with significant cardiovascular pathology such as cardiac hypertrophy, inflammation, pro-fibrotic, and atherogenic markers and aortic calcification in an animal model as assessed 1 month post exposure. Antioxidant supplementation prevented cardiac hypertrophy and attenuated indicators of atherosclerosis markers associated with cigarette smoke exposure.

## Introduction

Cardiovascular diseases are the leading causes of mortality and morbidity for men and women worldwide (Lim et al., 2012; Heron, 2013). In USA, one person will die every 37 s due to cardiovascular pathology. In developing countries, cardiovascular diseases are creating worsening financial and socioeconomic burden due to a steady increase in its prevalence and associated cost (Lloyd-Jones et al., 2009; WH Organization, 2011). Clinical studies documented a significant link between cardiovascular diseases and modifiable risk factors that include combustible tobacco smoking (C.f.D.C.a.P., (US);, N.C.f.C.D.P.a.H.P. (US);, and O.o.S.a.H. (US), 2010). Cigarette smoking is the main form of tobacco smoking and its addictive habit has increased the number of smokers, worldwide, to almost a billion individual (Ng et al., 2014; Eriksen et al., 2015). It is the burning process of tobacco in cigarettes that releases nicotine, and unfortunately, accompanied with bulk of chemicals that are either toxic or carcinogenic to the human body (Rodgman and Perfetti, 2012).

The correlation between cardiovascular diseases and cigarette smoking can be attributed to an increase in oxidative stress due to excessive generation and inhalation of free radicals from the combustion of tobacco and other contaminants (Pryor and Stone, 1993). Free radicals can be generated *in vivo*; however, protective mechanisms in the human body strictly regulate their concentration levels by maintaining a well-balanced buffer system (Dröge, 2002). Once the buffer system is overwhelmed by exogenous free radicals inhaled from cigarette smoke, an overall oxidative stress status is observed. In addition, oxidative stress will lead to the initiation of atherosclerosis by reducing protective nitric oxide bioavailability coupled with the activation of the adhesion of macrophages to endothelial cells causing detrimental effects on endothelial cell cytoskeleton (Cai and Harrison, 2000; Mercado and Jaimes, 2007; Kietadisorn et al., 2012). Harrison et al and others demonstrated an association between the intensity of oxidative stress and the extent and severity of coronary artery disease, vascular disease development, atherosclerosis and hypertension (Harrison et al., 2003; Vassalle et al., 2004). Furthermore, oxidative stress is also implicated in various other illnesses that include autoimmune diseases, malignancy, emphysema, and chronic obstructive lung disease (Mózsik et al., 1992; Brownlee, 2001). Various drinks and fruit juices can be a great source of different exogenous antioxidants due to their high content of polyphenols. Pomegranate juice is recognized for its antioxidant, anti-inflammatory, anti-atherogenic, and anti-tumorigenic properties (Gil et al., 2000; Lansky and Newman, 2007). Pomegranate has very high antioxidant activity, which is due to high content of polyphenols (Guoa et al., 2003; Li et al., 2006; Basiri, 2015), anthocyanins, tannins, and lipoic acid (Vroegrijk et al., 2011). Moreover, punicalagin and punicalin are found in pomegranate and they hydrolyzed to ellagic acid which has high antioxidant activity (Gil et al., 2000). The combination of punicalagins with phytochemicals such as ellagic acid synergistically enhances pomegranate juice superior antioxidant properties when compared to other polyphenol rich juices or beverages (Longtin, 2003). In this study, we have assessed the damaging effects of cigarette smoking exposure on the cardiovascular system and examined whether supplementation of antioxidants will attenuate the effects of cigarette smoking in an animal model. To our knowledge, this work highlighted the effect of cigarette smoking on the vascular system mediated via the upregulation of bdkrb1 levels in mediating the pathology of smoke while pomegranate supplementation reversed the smoking-induced effects on the kinin receptors. These effects were coupled by a decrease in the aortic calcification after pomegranate supplementation which further highlights more on the effects of smoke-mediated pathology on the cardiovascular system.

## Materials and methods

### Rats and cigarette smoking

This study was approved by the Institutional Animal Care and Use Committee (IACUC) of the American University of Beirut (AUB) with the following approval number **14-04-296**. Two-month old Male Sprague-Dawley rats weighing 250 g, originally purchased from Charles River Laboratories (Wilmington, MA, USA), were inbred and housed with controlled temperature and humidity at the AUB animal care facility. Rats had sterile bedding and *ad-libitum* access to water and rodent feed was provided. At the end of the experiment, animals were anesthetized with isoflurane and euthanized by cervical dislocation. Hearts were dissected, weighed and heart weight to body weight (H/B) ratio was calculated. Aorta samples were snap frozen in liquid nitrogen then stored at −80°C for Immunofluorescence, calcification, RNA isolation and protein analyses.

Forty eight animals were divided into four groups: (control), (cigarette smoking exposed-CS), (cigarette smoking exposed + antioxidant-AO-pomegranate supplemented group) and (antioxidant-AO-pomegranate supplemented group) and each group consisted of twelve animals (4 for RNA extraction, 4 protein extraction and 4 for immunohistochemistry). Cigarette smoke exposure apparatus (ONARES, CH Technologies, USA) included a smoke generator with a mixing/conditioning chamber and a “nose only” rodent exposure carousel. Animals were adapted to retainers for 1 week prior to initiating room air or cigarette smoke exposure as depicted in Supplementary Figure 1. Rats were then positioned in retainers and placed into the holes of the carousel. Animals received a continuous flow of cigarette smoke or room air into the airways via the “nose only” delivery system. As described before, the smoking rate was controlled to one puff of smoke each minute (Husari et al., 2016). Rats of the CS and CS + AO groups were exposed to cigarette smoke generated from 3R4F cigarettes (University of Kentucky, Lexington, KY, USA), which are scientifically prepared cigarettes concentrated with toxins and chemical rendering the study timeline suitable to observe the effects of smoking on the rats (Roemer et al., 2014). The cigarette smoke exposure was performed over two daily sessions (9:00 am and 2:00 pm) for 5 days per week and each session lasted for 1 h. On the other hand, rats of the control and AO groups were placed in the carousel but received room air. The total duration of the experiment was 1 month (refer to Supplementary Figure 1).

### Pomegranate juice as antioxidant supplementation

The antioxidant (AO) utilized in this study was pomegranate juice concentrate (Wonderful Variety, POM Wonderful, LA, USA). AO and CS + AO groups received pomegranate supplementation, while control and CS received placebo (regular water). Pomegranate juice supplementation to AO groups was started 1 week prior to cigarette smoke at room air exposure and was maintained throughout the experiment. Animals received 80 μM of polyphenols /ml/day of pomegranate juice. The pomegranate juice dose was prepared daily and mixed with the drinking water. Daily fluid intake was observed for all animals. The other groups received placebo free drinking water. The dose of pomegranate juice supplementation was deduced from previous studies performed by our group (Husari et al., 2014, 2016).

### Body weight and blood pressure measurements

Body weights and blood pressure were measured and recorded weekly. Blood pressure was measured by using the tail-cuff method (Kent scientific, Torrington, Connecticut, USA). Each session consisted of 5 acclimatization cycles and followed by 10 blood pressure measurement cycles. Systolic and diastolic blood pressures were recorded and charted. Average of the 10 readings was averaged for each animal.

### Dihydroethidium (DHE) staining protocol

Levels of reactive oxygen species (ROS) were assessed using the Dihydroethidium (DHE) staining method (Calbiochem, Darmstadt, Germany). Briefly, 10 μM of DHE were applied to aorta sections and incubated in a light protected humidified chamber for 15 min at 37°C, followed by washing with PBS. Nuclei of the tissues were stained by Bisbenzimide (Hoechst 33342 stain, Sigma-Aldrich, Taufkirchen, Germany). Images of tissues were acquired using Laser Scanning Confocal Microscope (Leica microsystems, Cambridge, UK). Zen 2011 was used to quantify the intensity of the Fluorescence of DHE. Control was used as a reference.

### RNA isolation and real-time PCR protocol

Changes in the mediators' transcriptional levels were assessed using Reverse Transcriptase-Polymerase Chain Reaction (RT-PCR); RNA was extracted using the TRIzol method (Invitrogen, Carlsbad, CA, USA). Briefly, 1 ml of TRIzol reagent was used per 50–100 mg of tissue sample, grinded with liquid nitrogen and followed by chloroform extraction. RNA samples were precipitated and stored at −80°C for further use. RNA was quantified using a 260/280 nm absorbance ratio method. Total RNA (1 μg) was reverse-transcribed into first strand cDNA by iScript cDNA Synthesis Kit (Bio-Rad laboratories, Hercules, CA, USA). Real time-PCR was performed using the CFX96 system (Bio-Rad laboratories, Hercules, CA, USA) with SYBR® Green JumpStart™ Taq ReadyMix (Sigma-Aldrich, Taufkirchen, Germany). Specific primers (Tib-Molbiol, Berlin, Germany) were used to assess the expression of mediators in tissues (*IL-1*β: Fp CACCTCTCAAGCAGAGCACAG, Rp GGGTTCCATGGTGAAGTCAAC; Tumor necrosis factor (TNF-α): Fp AATGGGCTCCCTCTCATCAGTTC, Rp TCTGCTTGGTGGTTTGCTACGAC; Fibronectin (Fn1): Fp CCACAGCCATTCCTGCGCCA and Rp: TCACCCGCACTCGGTAGCCA; Leptin receptor (ObR): Fp TGACCACTCCAGATTCCACA and Rp: CCACTGTTTTCACGTTGCTG). PCR products and their corresponding melting temperatures were analyzed using the CFX96 manager software (Bio-Rad laboratories, Hercules, CA, USA). Correction for loading was achieved by subtracting for local background and normalizing against the cDNA levels of the GAPDH housekeeping gene (GAPDH: Fp GTATTGGGCGCCTGGTCACC, Rp CGCTCCTGGAAGATGGTGATGG).

### Immunohistochemistry of bradykinin receptor 1 and bradykinin receptor expression

Immunohistochemistry (IHC) staining was utilized to assess levels of Bradykinin receptor 1 (Bdkrb1) and Bradykinin receptor 2 (Bdkrb2) expression in aorta sections among the different study groups. Briefly, 5 μm frozen aortic sections were prepared using Cryostat Microtome (Leica Biosystems, Wetzlar, Germany) and stored at −80°C freezer for immunohistochemistry staining. Slides were stained with antibodies against either Bdkrb1 (sc-25484, Santa-Cruz, Dallas, Texas, USA) or Bdkrb2 (ab101704, Abcam, Cambridge, MA, USA) for 1 h at room temperature, then washed 4 times with PBS for 5 min each. Slides were then incubated with anti-Rabbit conjugated to Alexa fluor 647 (Abcam, Cambridge, MA, USA) for 1 h at room temperature, then washed 4 times with PBS for 5 min each. Nuclei of the tissues were stained by Bisbenzimide (Hoechst 33342 stain, Sigma-Aldrich, Taufkirchen, Germany). Images of tissues were acquired using Laser Scanning Confocal Microscope (Leica microsystems, Cambridge, UK). Zen 2011 was used to quantify the intensity of the Fluorescence of Bdkrb1 or Bdkrb2. Control was used as a reference.

### Protein extraction and western blotting

Samples from aortas were crushed in liquid nitrogen and homogenized in 1X RIPA buffer (250 mM Tris-HCL, 750 mM sodium chloride, 5% Igepal CA-630, 5% sodium deoxycholate and 0.5% sodium dodecyl sulfate) containing the following inhibitors (1 mM PMSF, 1 mM benzamidine, 2 mM Na Orthovanadate, 10 mM NaF, 1 mM Na pyrophosphate, 2 μg/ml Leupeptin, 2 μg/ml Aprotinin and 1 μg/ml DTT). This was followed by centrifugation at 12000 × g for 10 min at 4°C. Total protein concentration was then determined using Bio-Rad Protein assay kit II (Bio-Rad, Hercules, CA, USA). 40 μg of protein samples were separated in 10% polyacrylamide gel (29:1 acrylamide:bis) and then transferred to nitrocellulose membrane. Membranes were blocked with 5% skimmed milk in TBST1x solution for 1 h at room temperature (RT). Membranes were incubated with primary antibody solutions against Bdkrb1 (sc-25484, Santa-Cruz, Dallas, Texas, USA), Bdkrb2 (ab101704, Abcam, Cambridge, MA, USA), Fn1 (ab23750, Abcam), ObR (ab5593, Abcam), or β-actin (ab8226, Abcam) overnight at 4°C, then followed with 4 washes with 1x TBST and incubated with HRP-conjugated anti-rabbit or anti-mouse (only for β-actin) solutions for 1 h at (RT). Chemiluminescence was activated by Amersham ECL Western Blotting Detection Reagent (GE Healthcare Bio-Sciences, Pittsburgh, PA, USA) and acquired by ChemiDoc™ MP system (Bio-Rad, Hercules, CA, USA). Intensity of bands was then determined by densitometry, using ImageJ software.

### Von kossa staining protocol

The mineralization capacity of aortas, a feature largely attributed to osteoblasts, was confirmed by performing Von Kossa staining. Aorta slides were fixed for 1 h on ice with 70% ethanol and washed three times with ultrapure water. Slides were then stained in the dark with 2% silver nitrate solution for 30 min (Merck Corporate, Kenilworth, USA), washed three times with ultrapure water and left under ultraviolet light for 1 h. Finally, slides were counterstained with 0.1% eosin (Sigma-Aldrich, Taufkirchen, Germany) and images were acquired with light microscope.

### Statistical analysis

Results were expressed as individual data or as the mean ± SEM. Statistical comparisons were performed using One-way ANOVA (analysis of variance), followed by Bonferroni correction method with an “all pairwise comparison” in order to determine statistical significance. The *p*-value was determined and values for *p* < 0.05 was considered significant. SigmaStat 3.1 software were used to perform statistical analysis.

## Results

### Effect of smoking on body weight and blood pressure

In order to investigate the effect of smoking on the rat cardiovascular parameters, we measured body weight and blood pressure of the different smoke exposed, AO supplemented and control groups. There was no significant change in the weight of animals between the different study groups throughout the experiment. The effect of smoking on rats' blood pressure (systolic and diastolic) did not commence to any significant unidirectional or bidirectional changes in CS group when compared to control group (data not shown; data were collected and analyzed however no significant changes were observed).

### The antioxidant effect of pomegranate juice on smoking-induced ROS production

**Figure 1:**
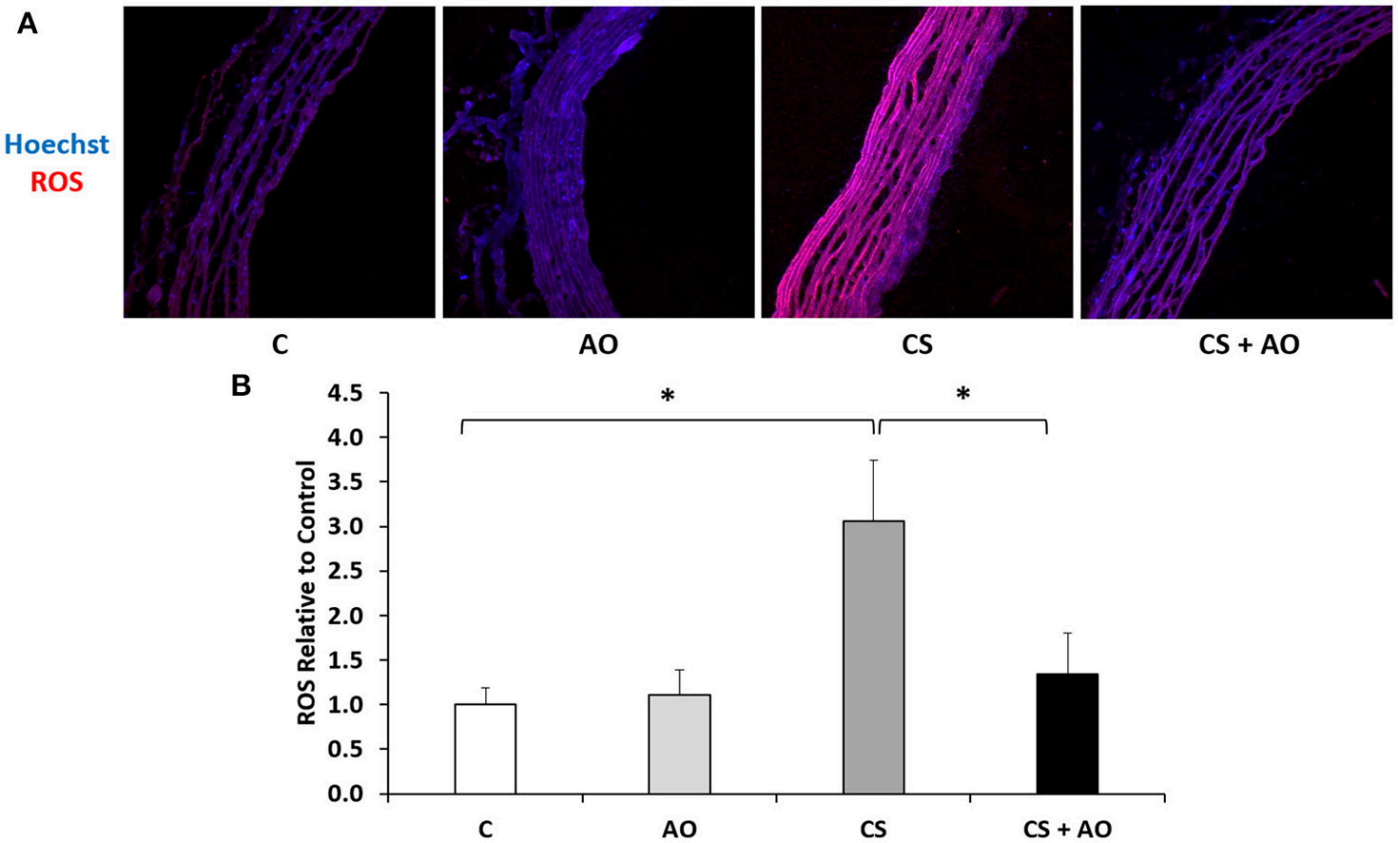
**Reactive oxygen species assessment post experimental smoke. (A)** AO attenuates CS induced ROS formation. Notes: 5 μm thick sections mounted on microscope slides were incubated with DHE. ROS levels were induced after CS and were attenuated in CS + AO. Images captured using 40X magnification. **(B)** Quantification of the intensity of ROS formation. Intensity of ROS staining was determined from ZEN software and normalized to the Hoechst level relative to the Control samples. CS group had a significantly higher ROS to Hoechst ratio compared to control. ROS was significantly reduced in CS + AO in comparison with CS. One Way Anova test was used to check for significance between the groups. Error bars represent SE. Asterisks indicate statistically significant associations (*P* < 0.05). CS, cigarette smoke; ROS, reactive oxygen species; AO, antioxidant.

Oxidative stress analysis was assessed via performing DHE staining that evaluates the generation of superoxide as major component of the ROS. DHE staining of aortic tissue among the different groups revealed a significant increase in ROS levels in CS group compared to the control group (*p* < 0.001) indicative of an altered oxidative stress status (Figures 1A,B). Supplementation of pomegranate juice significantly reversed the observed ROS generation (*p* < 0.001; Figures 1A,B).

### Effect of smoking on heart to body weight and associated fibrotic marker expression (ObR & Fn1)

**Figure 2:**
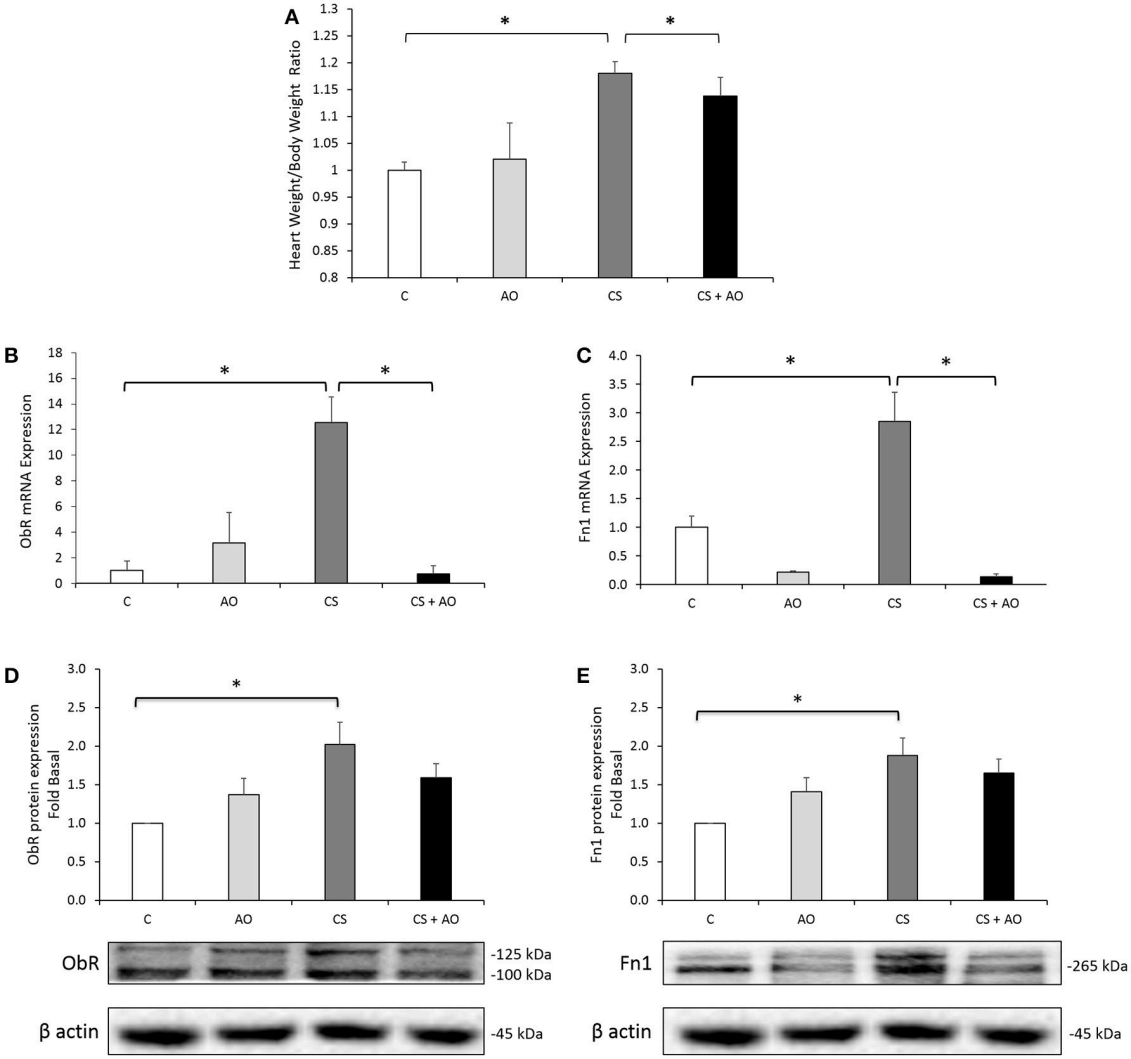
**(A)** Heart to Body weight ratio (H/B) after 4 weeks of CS. Mean Heart to Body weight ratio (H/B) compared to control. CS group had a significantly higher H/B ratio compared to control. H/B ratio was significantly reduced in CS + AO in comparison with CS. Error bars represent SE. One Way Anova test was used to check for significance between the groups. Asterisks indicate statistically significant associations (*P* < 0.05). **(B,C**) AO reduces transcriptional expression of fibrotic (ObR and Fn1) markers in rat aortas. Transcriptional expression of **(B)** ObR and **(C)** Fn1 were induced in the aortas of CS group. AO reduced the smoking-induced gene expression of ObR and Fn1. Data on each target mRNA was normalized to GAPDH. One Way Anova test was used to check for significance between the groups. Error bars represent SE. Asterisks indicate statistically significant associations (*P* < 0.05). **(D,E)** AO reduces protein expression of fibrotic (ObR and Fn1) markers in rat aortas. Protein expression of **(D)** ObR and **(E)** Fn1 were induced in the aortas of CS group. Data on each protein was normalized to β-actin. Error bars represent SE. One Way Anova test was used to check for significance between the groups. Asterisks indicate statistically significant associations (*P* < 0.05).

Heart to body weight ratio was performed on the different groups indicative of heart hypertrophy. A statistically significant increase in the heart to body weight ratio was observed in the CS group compared to the control group (*p* = 0.007; Figure 2A) which was decreased significantly by the administration of pomegranate juice in the CS + AO group (*p* = 0.016; Figure 2A).

Along the same line, the transcriptional expression of ObR and Fn1 was significantly upregulated in aortas of CS group by more than 12-fold and 3-fold (both with *p* < 0.001); respectively, (Figures 2B,C). On the protein level, these results were validated using Western blot analysis (Figures 2D,E); we have shown that cigarette smoke induced significantly the upregulation of protein expression of ObR and Fn1 proteins (*p* = 0.041 and *p* = 0.043); respectively (Figures 2D,E) similar to what was observed on the transcription mRNA level. Pomegranate juice supplementation significantly reduced cigarette smoking (CS) induced gene/protein expression of ObR and Fn1 in CS + AO group (*p* < 0.001) as depicted in Figures 2B–E.

### Effect of smoking on aortic calcification

**Figure 3:**
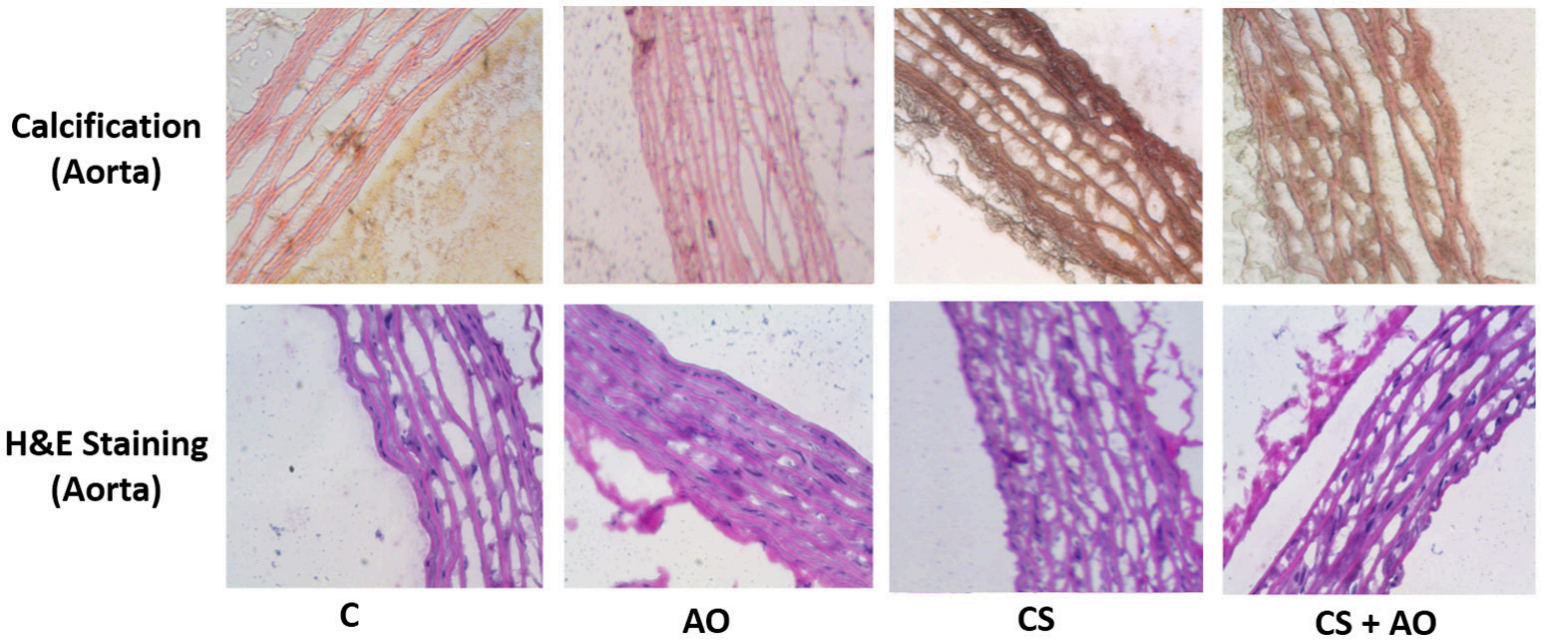
**Smoking-induced aorta calcification is stopped by AO**. The effect of CS on calcification in rat aortas, was assessed using von Kossa staining. CS induced calcium deposition in the aortas, which was significantly reduced with AO supplementation in (CS +AO) group. All pictures were taken at 40X magnification. For interpretation of Von Kossa stain, calcium salt in mass deposits appeared black, calcium in dispersed deposits appeared gray, nuclei appeared red, and cytoplasm appeared light pink.

Von Cossa staining, a measure of tissue calcium deposition, was employed to assess levels of calcification in the aorta of different groups. Data observed showed an increase in the aortic calcification in the CS group compared to control group (Figure 3) and; interestingly, AO supplementation prevented aortic calcification in the CS + AO group.

### Effect of smoking on expression of Bdkrb1 and Bdkrb2

**Figure 4:**
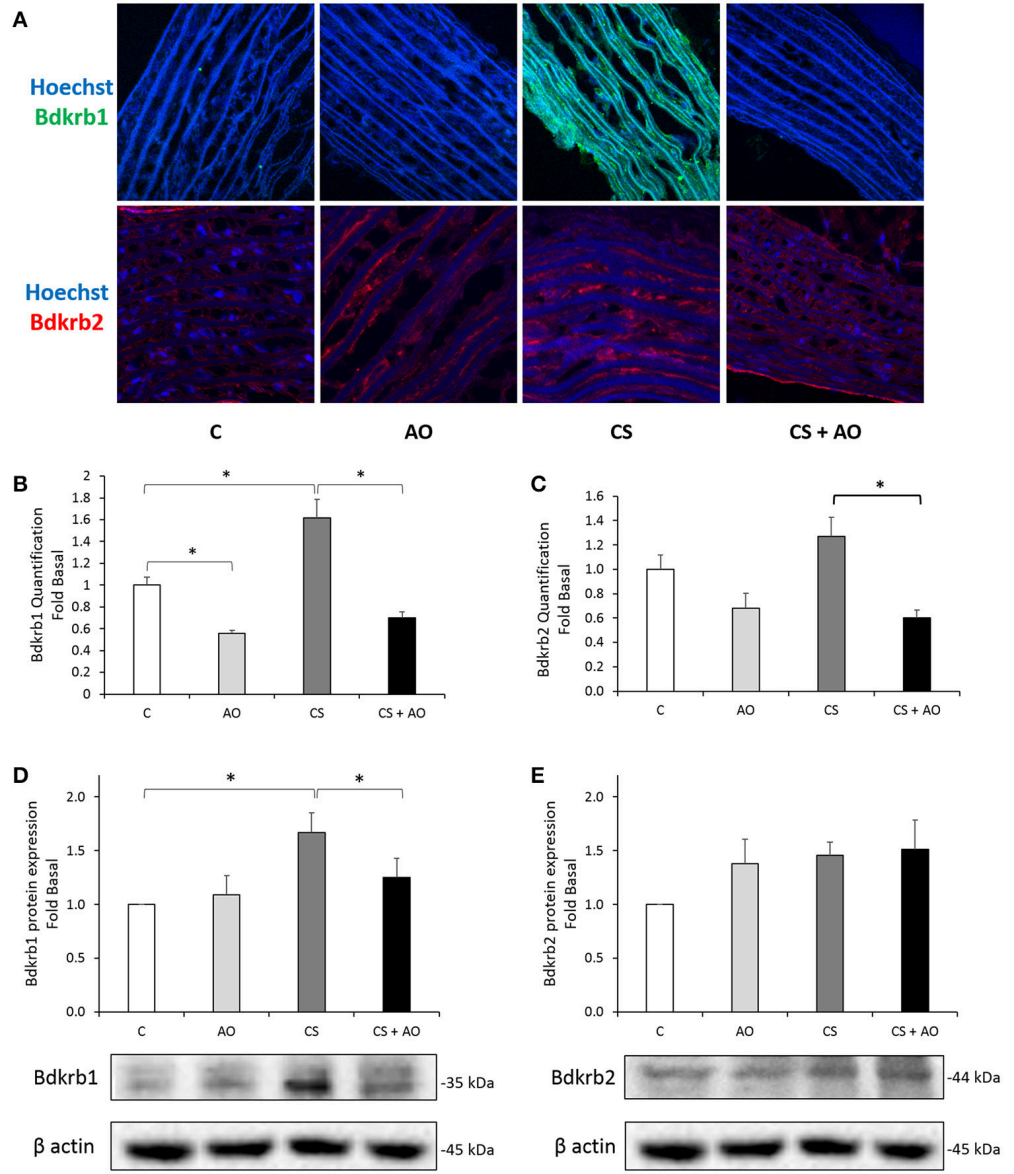
**Assessment of Bdkrb1 and Bdkrb2 in the aortas of rat tissues. (A)** Immunohistochemistry staining for Bdkrb1 and Bdkrb2 in the aortas of rat tissues. Bdkrb1 protein expression is induced in the aortas of smoking (CS) group. CS-induced expression was abolished in the presence of AO in the (CS + AO). All pictures were taken at 40X magnification. **(B)** Quantification of the intensity of the immunofluorescence of Bdkrb1. Intensity of Bdkrb1 staining was determined from ZEN software and normalized to the Hoechst level relative to the Control samples. CS group had a significantly higher Bdkrb1 to Hoechst ratio compared to control. Bdkrb1 staining was significantly reduced in CS + AO in comparison with CS. Error bars represent SE. One Way Anova test was used to check for significance between the groups. Asterisks indicate statistically significant associations (*P* < 0.05). **(C)** Quantification of the intensity of the immunofluorescence of Bdkrb2. Intensity of Bdkrb2 staining was determined from ZEN software and normalized to the Hoechst level relative to the Control samples. Bdkrb2 staining was significantly reduced in CS + AO in comparison with CS. Error bars represent SE. One Way Anova test was used to check for significance between the groups. Asterisks indicate statistically significant associations (*P* < 0.05). **D** and **E**: protein expression assessment of the Bdkrb1 and Bdkrb2 in rat aortas. Protein expression of **(D)** Bdkrb1, but not **(E)** Bdkrb2, was induced in the aortas of CS group. AO significantly reduced the expression of Bdkrb1 in the CS + AO group. Data on each protein was normalized to β-actin. Error bars represent SE. One Way Anova test was used to check for significance between the groups. Asterisks indicate statistically significant associations (*P* < 0.05).

Due the major contributing role of kinin receptors (*Bdkrb1 and Bdkrb2*) on inflammation and oxidative stress mechanisms, protein expression of Bdkrb1 and Bdkrb2 was assessed by IHC staining and Western blot analysis. Our data indicated that cigarette smoking significantly increased the protein expression of Bdkrb1; [IHC and Western blotting] (*p* < 0.001, *p* = 0.026; respectively); which was not observed with Bdkrb2 as shown in Figure 4. On the other hand, Bdkrb1 protein levels were shown to decrease upon pomegranate supplementation in the CS + AO group (*p* < 0.001; Figure 4).

### Effect of smoking on the inflammatory mediators IL-1β and TNF-α

**Figure 5:**
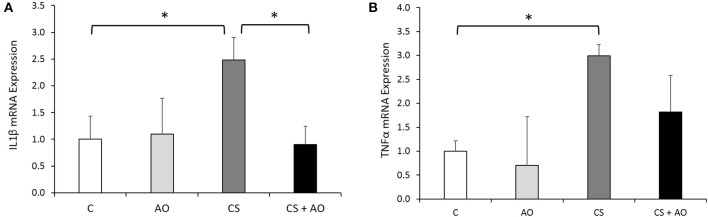
**AO reduces transcriptional expression of inflammatory (IL1β, TNF-α) in rat aortas**. Transcriptional expression of **(A)** IL-1β, and **(B)** TNF-α were induced in the aortas of CS group. AO reduced the smoking-induced gene expression of IL-1β. Data on each target mRNA was normalized to GAPDH. Error bars represent SE. One Way Anova test was used to check for significance between the groups. Asterisks indicate statistically significant associations (*P* < 0.05).

Coupled to the levels of kinin level assessment, inflammatory mediators including IL-1β and TNF-α were evaluated by RT-PCR. The expression of IL-1β and TNF-α was significantly increased in CS group compared to control group (*p* = 0.022 and 0.048; respectively). Pomegranate juice supplementation to CS group significantly reduced the expression of IL-1β (*p* = 0.024). Although the TNF-α levels were reduced in CS + AO group but the reduction did not commence to be statistically significant (Figures 5A,B).

## Discussion

Smoking is a worldwide public health burden and is associated with detrimental clinical problems, impacting the cardiovascular and the pulmonary systems. Cardiovascular disease is the leading cause of death worldwide (Kadhum et al., 2015; Morris et al., 2015). Many studies have reported a strong association between smoking and cardiovascular disease occurrence (Doria et al., 2003; Banderali et al., 2015; Morris et al., 2015; Zaquia Leão et al., 2016). Nevertheless, other studies have failed to find the association between smoking and cardiovascular disease, which is possibly due to the study population and size (Akhtar and Asghar, 2015). Although the exact mechanisms by which smoking promote cardiovascular disease are still under investigation, many studies have reported that smoking induces cardiovascular injury, elevates inflammation, and leads to increase in the reactive oxygen species (ROS) generation (Talukder et al., 2011; Jaffa et al., 2012).

In this work, rats were exposed to 1 month cigarette smoke and were assessed for pathophysiologic cardiovascular remodeling indices including cardiac hypertrophy, inflammatory marker and ROS generation. These endpoints were evaluated upon pomegranate supplementation as a source of antioxidants. Data from this work indicated that smoke exposure was associated with significant cardiovascular remodeling events as discussed later. In our model, the “nose only” delivery paradigm, which mimics primary smoking exposure, delivered cigarette smoke directly into the airways of tested animals and eliminated inadequate cigarette smoke exposure secondary to whole-body exposure. Our smoke exposure model was previously employed to assess different pulmonary events post smoke exposure (Raza et al., 2013; Husari et al., 2014).

In our study, an increase in oxidative stress was observed in the aortas of CS groups as shown with the DHE staining; on the other hand, pomegranate juice supplementation reduced this elevated oxidative stress status and attenuated cardiac hypertrophy; thus, providing further evidence for the injurious role of oxidative stress on the cardiovascular system (Figure 1). Pomegranate has very high antioxidant activity, which is due to high content of polyphenols (Guoa et al., 2003; Li et al., 2006; Basiri, 2015), anthocyanins, tannins and lipoic acid (Vroegrijk et al., 2011). Moreover, punicalagin and punicalin are found in pomegranate and they are hydrolyzed to ellagic acid which has high antioxidant activity (Gil et al., 2000). It is very important to know that punicalagin is a unique substance to pomegranate. Antioxidant activity feature of pomegranate is important since it has several important biological properties and it can be considered as potential and therapeutic substance in attenuating the damaging effects of cigarette smoking.

Similarly, our data indicated that cigarette smoke exposure was associated with an increase in heart to body weight ratio suggestive of cardiac hypertrophy (Figure 2A). Similar findings of cardiac hypertrophy and cardiac remodeling secondary to cigarette smoking were noted in previous studies (Talukder et al., 2011; Minicucci et al., 2012). Talukder et al showed that cigarette smoking caused hypertension, cardiac hypertrophy and remodeling in animals exposed to cigarette smoke (Talukder et al., 2011). Talukder attributed the cardiovascular remodeling findings to an increase in oxidative stress status in cardiovascular system secondary to cigarette smoking (Talukder et al., 2011). Taken together, increased ROS levels and oxidative stress are significant findings in cardiac hypertrophy secondary to cigarette smoking.

Post hypertrophic and oxidative stress assessment, we targeted fibrotic/atherogeneic–related markers (Fn1 and ObR) as contributing factors involved in the remodeling process post smoke exposure. In this study, we have shown that the expression of Fn1 and ObR in the aortas were significantly higher in CS group. Indeed, cigarette extract is known to increase the release of Fn1 from fibroblasts isolated from the lungs of COPD patients (Krimmer et al., 2013). The role of Fn1 in pathological heart growth induced by several factors have been investigated (Crawford et al., 1994; Plante et al., 2004; Arslan et al., 2011). It has been shown that Fn1 gene expression correlates with cardiac remodeling induced by several factors such as volume overload (Plante et al., 2004; Konstandin et al., 2013), hypertension (Crawford et al., 1994) and myocardial infarction (Arslan et al., 2011) and most importantly, cardiomyocyte hypertrophy which is regulated via activation of Nuclear Factor of Activated T cells (NFAT; Konstandin et al., 2013). Moreover, lack of Fn1 expression significantly attenuated the physiological growth in cultured cardiac cell (Chen et al., 2004; Konstandin et al., 2013).

Furthermore, we investigated the role of ObR in mediating the smoke induced cardiac remodeling events where we demonstrated that smoking significantly increased ObR expression. Data from our laboratory have shown the involvement of leptin receptor activation on cardiomyocytes hypertrophy and remodeling (Zeidan et al., 2006). Moreover, it is demonstrated that ObR increases the expression of adhesion intercellular molecules and cyclooxygenase 2 on aorta tissue inducing endothelial dysfunction and vascular injury (Manuel-Apolinar et al., 2013). In our work, pomegranate juice supplementation attenuated the expression of Fn1 secondary to cigarette smoking and prevented the increased expression of ObR as demonstrated in Figures 2B,C; thus, limiting further cardiovascular injury. Our study showed the involvement of both Fn1 and ObR expression might contribute to the pro-hypertrophic response of smoking. This effect was attenuated by pomegranate juice supplementation (Figures 2B,C).

Furthermore, 1 month post cigarette smoke exposure, the CS group exhibited significant aortic calcification and vascular stiffening due to relentless injury to the aorta that was observed and reversed by pomegranate juice supplementation as shown in the Von Kossa staining shown in Figure 3. The observed calcification of the aorta of the CS group is in line with previous studies demonstrating this phenomenon (Szulc et al., 2014; Churchill et al., 2015; Ladich et al., 2016). However, to the best of our knowledge, we report to the first time that pomegranate juice supplementation would ameliorate the occurrence of the aortic calcification in experimental smoke paradigm (Figure 3). On the other hand, the mechanism by which aortic calcification is mediated is still under investigation. One study suggests that microvesicles and hydroxyapatite formation would be the initiation step of the aortic calcification (New and Aikawa, 2013; Aikawa et al., 2014). In addition, Aikawa et al has shown that the calcification site in the intimal region of the aorta occurs in a close proximity to inflammation sites (Aikawa et al., 2014). The latter mechanism could explain the observed decrease in the aortic calcification in our results, since pomegranate juice supplementation decreased the expression of many inflammatory markers as shown in Figures 4, 5. Having said that, further studies are warranted to decipher the mechanism(s) by which pomegranate modulate calcification process explaining the AO-smoking crosstalk. Furthermore, in this study, we have shown that cigarette smoke exposure is also associated with significant surge in the expression of inflammatory mediators (TNF-α, IL-1β) expressed in the aortic tissues coupled with an up-regulation of Bdkrb1 (Figures 4, 5). We have shown previously an elevation in the inflammatory pathways in lungs of CS group (Husari et al., 2014).

In addition, Bradykinin receptors 1 & 2 (Bdkrb1 and Bdkrb2) are known to mediate the inflammatory response of the lungs and other organs as well (Jaffa et al., 2012; Delemasure et al., 2013). Bdkrb1 is usually undetectable in healthy tissues, yet this receptor is strongly induced by the pro-inflammatory cytokine IL-1β cytokine pathway and oxidative stress via the transcriptional nuclear factor NF-κB (Lin et al., 2010; Krimmer et al., 2013). The up-regulation of Bdkrb1 and inflammatory mediators signifies aortic injury secondary to cigarette smoking. Interestingly, pomegranate juice supplementation attenuated the surge of IL-1β and prevented the induction of Bdkrb1 receptors in the aortas of CS group. We hypothesize that the inflammatory processes secondary to cigarette smoking may have triggered an associated increase in the expression of fibrotic/atherogenic markers.

Taken together, our work extends on previous published studies to decipher the mechanism(s) by which smoke exposure mediates cardia remodeling altering different cardiovascular parameters. Of interest, this study demonstrated that the administration of AO as pomegranate juice supplementation have ameliorated the occurrence of aortic calcification in post smoke paradigm. This was coupled with the smoke-induced upregulation of Bdkrb1 in the aortic tissue an observation that highlight the role of the kinin system involvement in smoke exposure and can be used as a potential therapeutic target as it was shown to be reversible by the administration of antioxidant post smoke exposure. Finally, although the results of the study show that pomegranate juice supplementation would ameliorate the detrimental effects of smoking on the cardiovascular system, the first and utmost precaution of smoking is abstaining smoking (primary or second hand ones).

## Conclusion

In this study, we dissected the different pathophysiological effects of cigarette smoking on the cardiovascular system in an animal model. In addition, we explored the role of a potent antioxidant on cigarette smoking-associated oxidative stress and subsequent harmful cardiovascular effects. The study showed that pomegranate juice supplementation attenuated the associated inflammatory, pro-fibrotic and atherogenic markers and prevented aortic calcification in an animal model. Results of this study suggest a promising impact of pomegranate juice on the well-being of cardiovascular system. Further animal and human studies are needed to explore the beneficial role of pomegranate juice in preventing cigarette smoking induced cardiovascular diseases.

## Ethics statement

This study was approved by the Institutional Animal Care and Use Committee (IACUC) of the American University of Beirut, with the following IACUC approval number 14-04-296. All experiments were conducted in compliance with current Good Clinical Practice standards and in accordance with relevant guidelines and regulations and the principles set forth under the Declaration of Helsinki.

## Author contributions

AH and FK conceptualized the study. MA, KZ, WF, FA, YH, NS, and EH performed experiments. AH, FK, and KZ contributed to the study design. MA, WF and KZ analyzed data. KZ, AH, and MA wrote the manuscript with input from the entire team. All authors reviewed and approved the manuscript.

### Conflict of interest statement

The authors declare that the research was conducted in the absence of any commercial or financial relationships that could be construed as a potential conflict of interest.

## Supplementary material

Supplementary Figure 1 **Design and timeline of the study**. Two months old Sprague Dawley rats were exposed to smoke exposure 1 week post acclimatization. Rats were exposed to 1 month smoke exposure paradigm. Pomegranate juice supplementation to AO group was started 1 week prior to cigarette smoke at room air exposure and was maintained throughout the experiment. Pomegranate supplementation was delivered by drinking bottles. All animals were sacrificed at 1 month time point.

## Abbreviations

CS: Cigarette smoking exposed
AO: Antioxidant-AO-pomegranate supplemented group
RT: Room temperature
ROS: Reactive oxygen species
TNF-α: Tumor necrosis factor.
