# Supplementary figures and images for: Cigarette Smoking-Induced Cardiac Hypertrophy, Vascular Inflammation and Injury Are Attenuated by Antioxidant Supplementation in an Animal Model
Source: Front Pharmacol. 2016 Nov 9;7:397. doi: 10.3389/fphar.2016.00397 (PMC5101594; doi:10.3389/fphar.2016.00397)

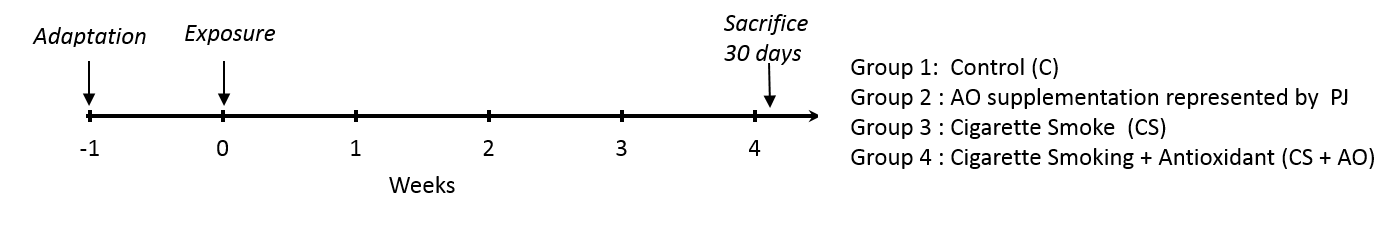

Supplement: Supplementary Figure 1 — Design and timeline of the study. Two months old Sprague Dawley rats were exposed to smoke exposure 1 week post acclimatization. Rats were exposed to 1 month smoke exposure paradigm. Pomegranate juice supplementation to AO group was started 1 week prior to cigarette smoke at room air exposure and was maintained throughout the experiment. Pomegranate supplementation was delivered by drinking bottles. All animals were sacrificed at 1 month time point. [file Image1.TIF]
